# A continuum of amorphous ices between low-density and high-density amorphous ice

**DOI:** 10.1038/s42004-024-01117-2

**Published:** 2024-02-20

**Authors:** Ali Eltareb, Gustavo E. Lopez, Nicolas Giovambattista

**Affiliations:** 1 Department of Physics, Brooklyn College of the City University of New York, Brooklyn, NY 11210 USA; 2 Ph.D. Program in Physics, The Graduate Center of the City University of New York, New York, NY 10016 USA; 3 Department of Chemistry, Lehman College of the City University of New York, Bronx, NY 10468 USA; 4 Ph.D. Program in Chemistry, The Graduate Center of the City University of New York, New York, NY 10016 USA

**Keywords:** Computational chemistry, Condensed-matter physics

## Abstract

Amorphous ices are usually classified as belonging to low-density or high-density amorphous ice (LDA and HDA) with densities *ρ*_*LDA*_ ≈ 0.94 g/cm^3^ and *ρ*_*HDA*_ ≈ 1.15−1.17 g/cm^3^. However, a recent experiment crushing hexagonal ice (ball-milling) produced a *medium*-density amorphous ice (MDA, *ρ*_*MDA*_ ≈ 1.06 g/cm^3^) adding complexity to our understanding of amorphous ice and the phase diagram of supercooled water. Motivated by the discovery of MDA, we perform computer simulations where amorphous ices are produced by isobaric cooling and isothermal compression/decompression. Our results show that, depending on the pressure employed, isobaric cooling can generate a continuum of amorphous ices with densities that expand in between those of LDA and HDA (briefly, intermediate amorphous ices, IA). In particular, the IA generated at *P* ≈ 125 MPa has a remarkably similar density and average structure as MDA, implying that MDA is not unique. Using the potential energy landscape formalism, we provide an intuitive qualitative understanding of the nature of LDA, HDA, and the IA generated at different pressures. In this view, LDA and HDA occupy specific and well-separated regions of the PEL; the IA prepared at *P* = 125 MPa is located in the intermediate region of the PEL that separates LDA and HDA.

## Abstract (web summary)

Recently, a medium-density amorphous ice (MDA) was synthesized via ball-milling of hexagonal ice, adding complexity to our understanding of the phase diagram of supercooled water and amorphous ice. Here, the authors use molecular dynamics simulations to show that, depending on the employed pressure, isobaric cooling can generate a continuum of amorphous ices with densities that expand in between those of low- and high-density amorphous ices, with some of these amorphous ices being remarkably similar to MDA.

## Introduction

Most substances exist in a single amorphous solid or glass state, with properties that vary smoothly with pressure and temperature. Water, however, is different. In 1984–1985, Mishima et al. showed that water can exist in two different amorphous solid states, low-density and high-density amorphous ice (LDA and HDA)^1,2^, at approximately *P* < 1 GPa and *T* < 135 K. LDA and HDA are remarkably different^3–7^; they are distinguishable to the naked eye^8^ and their densities differ by ≈ 20−25% (*ρ*_*LDA*_ ≈ 0.94 g/cm^3^ and *ρ*_*HDA*_ ≈ 1.15−17 g/cm^3^ at *T* = 77 K and *P* = 0.1 MPa)^2,9^. Perhaps more astonishing is the relationship of LDA and HDA. LDA and HDA can be interconverted by isothermal compression and decompression with properties that exhibit an apparent discontinuity during the LDA-HDA transformation, reminiscent of a first-order phase transition^2,10–13^.

A natural explanation for the existence of LDA and HDA is provided by the liquid-liquid phase transition (LLPT) hypothesis scenario proposed by Poole et al., and based on computer simulations of supercooled water^14^. In this scenario, water at low temperatures, in the supercooled regime, can exist in two different liquid states, low-density liquid (LDL) at low pressures and high-density liquid (HDL) at high pressures^15–17^. In the P-T phase diagram, LDL and HDL are separated by an LLPT line that ends at a liquid-liquid critical point (LLCP). The LLPT hypothesis scenario for water has received overwhelming support, particularly over the last few years, from both (i) computer simulations employing advanced free energy calculation techniques, and (ii) novel experiments that are able to access the properties of water on the nanosecond time scale^18–28^. Importantly, in the LLPT scenario, LDA and HDA are the glass counterpart of LDL and HDL, respectively^14,29–31^. Indeed, experiments show that LDA can be produced by hyperquenching liquid water at *P* = 0.1 MPa^32,33^, while isobaric cooling experiments of (emulsified) liquid water and aqueous solutions under pressure (*P* ≈ 0.3 − 0.5 GPa) produce an amorphous ice remarkably similar to HDA^34,35^.

At present, the most common view is that amorphous ices produced at *P* < 1 GPa can be classified as either LDA or HDA, with LDA/HDA representing *families* of amorphous ices^5,7^. Amorphous ices within the LDA and HDA families may differ slightly depending on the preparation process, but they exhibit similar densities and structural properties. However, this view has been challenged recently by the discovery of medium-density amorphous ice (MDA)^36^. In ref. 36, it is shown that ball-milling of hexagonal ice (*I*_*h*_) produces an amorphous ice, MDA, with a density (*ρ*_*MDA*_ = 1.06 g/cm^3^) and structure that are intermediate to those corresponding to LDA and HDA. While exciting, these novel experiments challenge our understanding of amorphous ice. The following questions naturally arise, what is the nature of MDA? is MDA related to the liquid state of water? can MDA be rationalized in the context of the LLPT scenario?

To address the questions raised above, we perform molecular dynamics simulations of a flexible water model (q-TIP4P/F) at low temperatures, in the supercooled liquid and amorphous ice states. In addition, we perform potential energy minimizations and Hessian calculations, and interpret the results in the context of the potential energy formalism (PEL)^37–39^. We find that isobaric cooling of liquid water at different pressures produces a continuum of intermediate amorphous ices (IA) with density and structure that are in between the corresponding density and structure of LDA and HDA. In particular, cooling at *P* = 125 MPa results in an IA with practically the same density and structure of MDA. Accordingly, our results indicate that MDA is not a unique structure in between LDA and HDA, and that it is not impossible that MDA is related to the liquid state of water via some thermodynamic path. Our PEL calculations suggest a simple interpretation of LDA, HDA, IA (and possibly MDA). In this view, LDA and HDA occupy well-separated regions of the PEL. IA produced by cooling at low-pressure (high-pressure) are located in the LDA (HDA) region of the PEL. Instead, IA produced at intermediate pressures (e.g., *P* = 125 MPa) are located in the intermediate region of the PEL, in between the regions associated to LDA and HDA.

## Results

### Intermediate amorphous ices produced by isobaric cooling

**Fig. 1:**
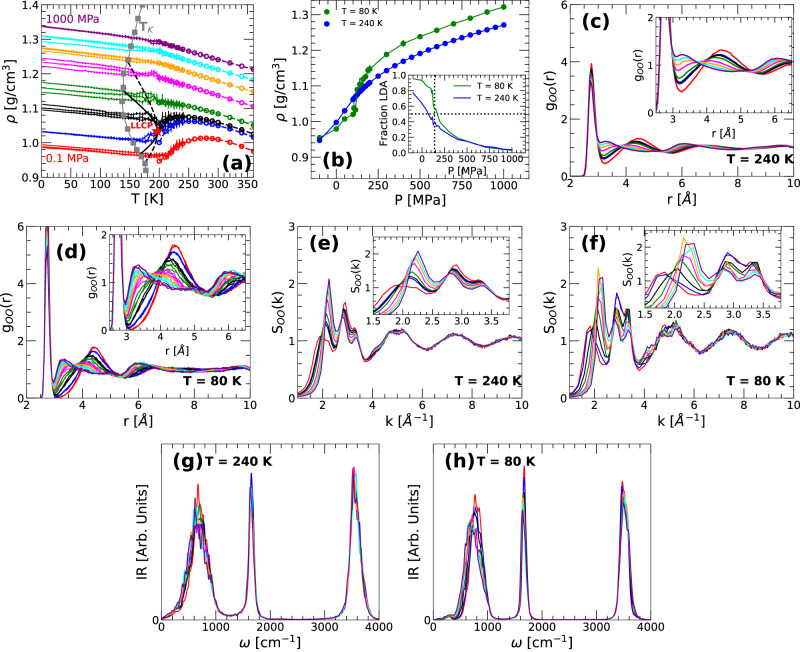
Thermodynamic, structural, and vibrational properties of water during vitrification (isobaric cooling) at different pressures. **a** Density of q-TIP4P/F water as function of temperature during isobaric cooling at *P* = 0.1, 100, 125, 200, 400, 600, 800, and 1000 MPa (dots, bottom-to-top); three independent runs are included for each pressure. Liquid water is first equilibrated at *T* = 240 K and it is then cooled at constant rate *q*_*T*_ = 10 K/ns to produce amorphous ice. Circles are the densities of liquid water in equilibrium at *T* ≥ 200 K. For comparison, also included are the LLCP (red star) and the associated binodal and spinodal lines (black dashed and solid lines, respectively). Solid gray squares represent the Kauzmann temperature *T*_*K*_(*P*) of q-TIP4P/F water from ref. 40. **b** Density of the equilibrium liquid at *T* = 240 K (blue line) and the corresponding amorphous ice at *T* = 80 K produced during the isobaric cooling runs included in (**a**). The amorphous ices produced by isobaric cooling at different pressures cover a continuum range of densities including the densities of LDA, MDA, and HDA. Inset: fraction of LDA molecules in the liquid and amorphous ice states included in the main panel. **c**, **d** OO RDF of water in the equilibrium liquid and amorphous ice states included in (**b**). **e**, **f** Structure factor *S*(*k*) of the liquid and amorphous ices included in (**c**, **d**), respectively. **g**, **h** Infrared spectra (IR) of the liquid and amorphous ices included in (**c**, **d**), respectively. Colors in (**c**–**h**) correspond to the same pressures indicated in (**a**). Insets in (**c**–**f**) are magnifications of the main panels.

Figure 1a shows the density of q-TIP4P/F water during isobaric cooling runs at selected pressures 0.1 ≤ *P* ≤ 1000 MPa (cooling rate *q*_*T*_ = 10 K/ns). For comparison, also included are the densities of q-TIP4P/F water in the equilibrium liquid state at *T* ≥ 200 K (circles). At all pressures studied, the density of the system during the cooling runs overlap with the corresponding equilibrium densities down to at least ${T}^{{\prime} }=200-210$ K, suggesting that equilibrium is lost upon cooling at $T\le {T}^{{\prime} }(P)$. It follows that the system looses equilibrium at temperatures slightly above the LLCP temperature of q-TIP4P/F water, *T*_*c*_ ≈ 197 K [*P*_*c*_ ≈ 162 MPa; red star in Fig. 1a]^40^. We note that water enters the amorphous ice state at slightly lower temperatures below *T*_*c*_. Indeed, for classical glasses, the density at low temperatures varies linearly with *T* (see refs. 31,41,42). It follows from Fig. 1a that, at the present cooling rate, the system vitrifies at approximately 160−200 K, depending on pressure. As one would expect, these temperatures are above the Kauzmann temperature *T*_*k*_(*P*) = 140−180 K (as calculated in ref. 40 using the PEL formalism^37–39^). At a given pressure, *T*_*k*_ defines the temperature at which the configurational entropy of the system is zero and hence, there is only one glass state available to the system at *T* ≤ *T*_*K*_.

One of the main points of Fig. 1a is that, by selecting appropriately the pressure for the cooling run, it is possible to generate a continuum of amorphous ices with intermediate densities between the densities of LDA and HDA; we will refer to such states as intermediate amorphous ices (IA). For example, at *T* = 80 K, the density of LDA obtained by cooling q-TIP4P/F water at *P* = 0.1 MPa is *ρ*_*LDA*_ = 0.98 g/cm^3^ while the density of HDA obtained by isobaric cooling at *P* = 1000 MPa is *ρ*_*HDA*_ = 1.32 g/cm^3^. As shown in Fig. 1b, the density of the IA obtained by cooling at 0.1 < *P* < 1000 MPa increases monotonically and continuously with increasing pressure, covering the densities *ρ*_*LDA*_ ≤ *ρ* ≤ *ρ*_*HDA*_; this density range includes, of course, the density of MDA, *ρ*_*MDA*_ = 1.06 g/cm^3^, reported in ref. ^36^. Our results in Fig. 1a, b are consistent with previous computer simulation studies of atomistic^42–44^ and coarse-grain^45^ water models showing that the densities of amorphous ices generated by isobaric cooling increase continuously with increasing pressure.

**Fig. 2:**
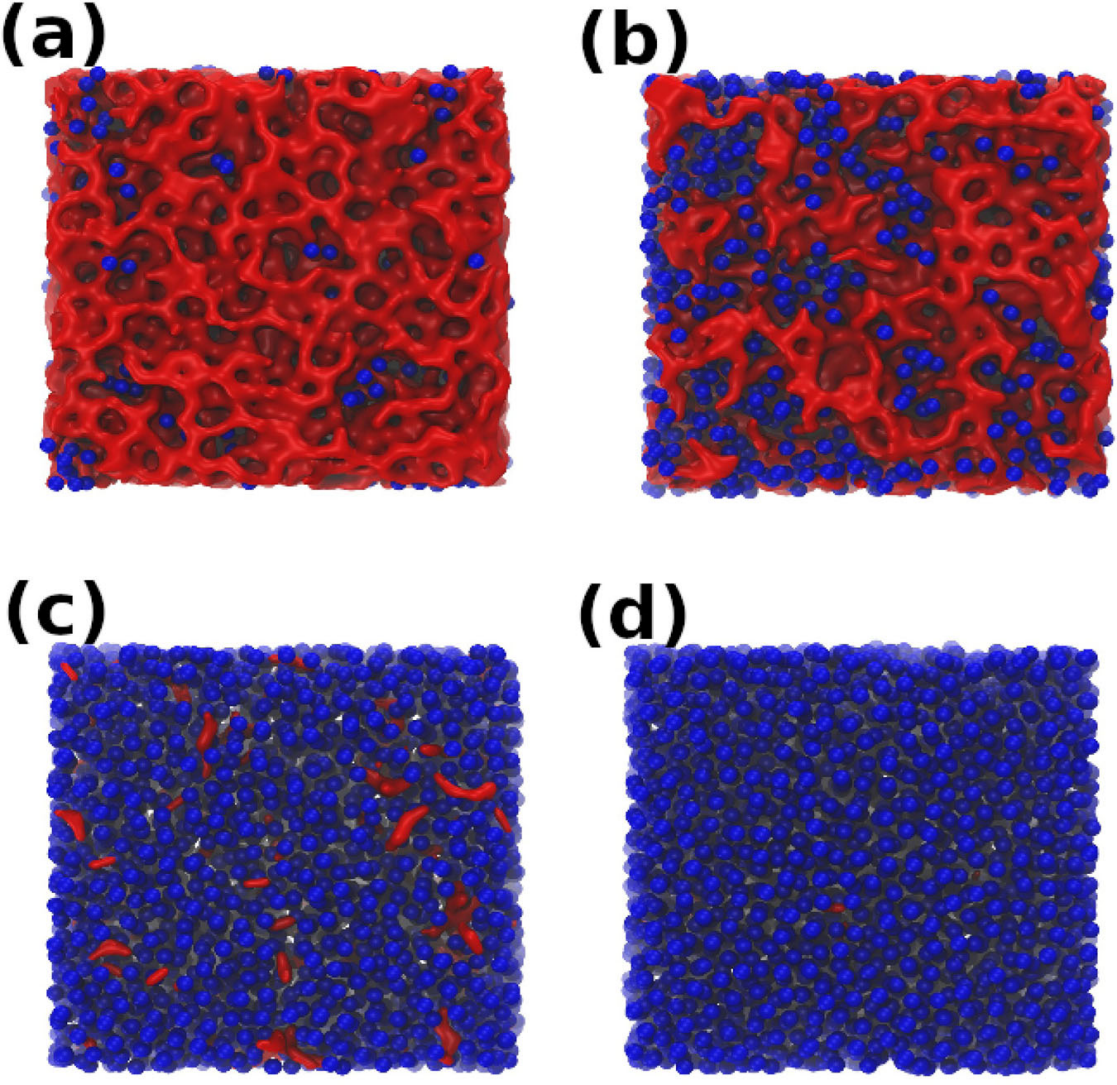
Snapshots of amorphous ices at *T* = 80 K prepared by isobaric cooling at different pressures. **a** *P* = 0.1 MPa, **b** *P* = 125 MPa, **c** *P* = 400 MPa, and (**d**) *P* = 800 MPa. The system is composed of *N* = 5118 water molecules. The oxygen atoms of the water molecules classified as HDA are shown as blue spheres; molecules classified as LDA are represented by a red surface. A molecule is classified as LDA (HDA) if its O atom has <5 (≥5) O nearest-neighbors within a distance *d*_*OO*_ = 3.4 Å. The IA do not show phase separation but resemble a mixture of LDA and HDA domains, with LDA (HDA) domains being the more dominant species at low (high) cooling pressures.

The intermediate amorphous ices (IA) obtained upon cooling at the pressures studied show no indication of phase-separation between LDA and HDA. However, as one would expect, the fraction of LDA (HDA) molecules in the IA decreases (increases) with increasing cooling pressure; see inset of Fig. 1b and Fig. 2. The change in the LDA/HDA composition of the IA with the cooling pressure is reflected in the structure of the corresponding amorphous ices. To show this, we include in Fig. 1c, d the oxygen-oxygen radial distribution function (OO RDF) of q-TIP4P/F water at *T* = 240 K, in the equilibrium liquid state, and at *T* = 80 K, in the glass state. At both temperatures, the increase in the number of HDA-like molecules with increasing pressures is accompanied with (i) a reduction of the OO RDF second peak at *r* ≈ 4.5 Å, and (ii) an increase of the OO RDF at *r* ≈ 3.0 − 3.5 Å, corresponding to the first interstitial space; (iii) the third maximum of the OO-RDF at *r* ≈ 6−6.5 Å also shifts towards smaller values of *r*. Importantly, points (i) and (ii) imply that, as the cooling pressure is increased, water molecules experience (on average) a displacement of their neighboring molecules from their second hydration shell toward their first hydration shell, filling up the corresponding first interstitial space. We note that the structural changes observed in our IA with pressure also occur during the LDA-to-HDA transformation induced by *compression* at low temperatures (see, e.g., ref. 5 and references therein). However, during the compression-induced LDA-to-HDA transformation, the LDA-to-HDA transition is sharp and hence, the structural changes are sudden and occur at a well-defined pressure (i.e., the interstitial space at *r* ≈ 3.0−3.5 Å is suddenly populated at such a specific pressure). Instead, the changes in the OO RDF of the IA evolve smoothly with pressure and hence, the interstitial space at *r* ≈ 3.0−3.5 Å is populated gradually as the cooling pressure is increased.

The OO RDF is not directly accessible to experiments. Instead, experiments have access to the structure factor of the liquid/glass, from which the OO RDF is calculated. Therefore, we include in Fig. 1e, f the structure factor *S*(*k*) of liquid/glassy water corresponding to Fig. 1c, d. As for the case of the OO RDF, the structural changes (with increasing cooling pressure) are much sharper in the IA than in the corresponding liquid states at *T* = 240 K. Importantly, the *S*(*k*) and RDF for the IA exhibit no signs of an isobestic point suggesting a continuous transformation of the IA, from LDA-like to HDA-like, with increasing cooling pressure^46^. This is also supported by the evolution of the location of the firs-peak of the *S*(*k*) with pressure, *k*_1_(*P*). As shown in Supplementary Fig. S2 (Supplementary Note 2), *k*_1_(*P*) increases monotonically and rather continuously with increasing pressure, from *k*_1_ ≈ 1.7−1.8 Å in the LDA state to *k*_1_ ≈ 2.1−2.3 Å in the HDA state (these values are consistent with experiments^18,19^). Overall, the behavior of *S*(*k*) for the IA (Fig. 1f), with increasing cooling pressure, is qualitatively similar to the continuous changes in the *S*(*k*) observed in the experiments of ref. 46 during the heating-induced uHDA-to-LDA transformation at 1 bar (uHDA refers to unannealed HDA).

The infrared spectra of LDA and HDA are also accessible to experiments^47^. The IR spectra of the liquids and IA included in Fig. 1c, d are shown in Fig. 1g, h. In both cases, there are only minor changes with pressure in the IR spectra of the IA over the stretching (*ω* ≈ 3500 cm^−1^) and bending (*ω* ≈ 1700 cm^−1^) frequency bands (see magnifications of Fig. 1h reported in Supplementary Fig. S3). The corresponding IR peaks decrease slightly with increasing cooling pressure, with a small shift in frequency. As shown in Supplementary Fig. S3 (Supplementary Note 3), the changes in the IR spectra of the IA shown in Fig. 1h in the OH stretching region [*ω* > 3000 cm^−1^] are in qualitative agreement with the IR spectra of LDA and HDA reported in the experiments of refs. 48,49. Most of the changes in the IR spectra occur in the librational band (*ω* < 1000 cm^−1^). In the case of the IA, the librational band peak becomes slightly smaller with increasing pressure and shifts towards lower frequencies. This shift to lower frequencies occurs as the IA evolves from LDA-like to HDA-like and hence, it seems to be related to the weakening of the hydrogen bond network (as the IA becomes less tetrahedral).

### Comparison of the Intermediate Amorphous Ices with LDA, MDA, and HDA

**Fig. 3:**
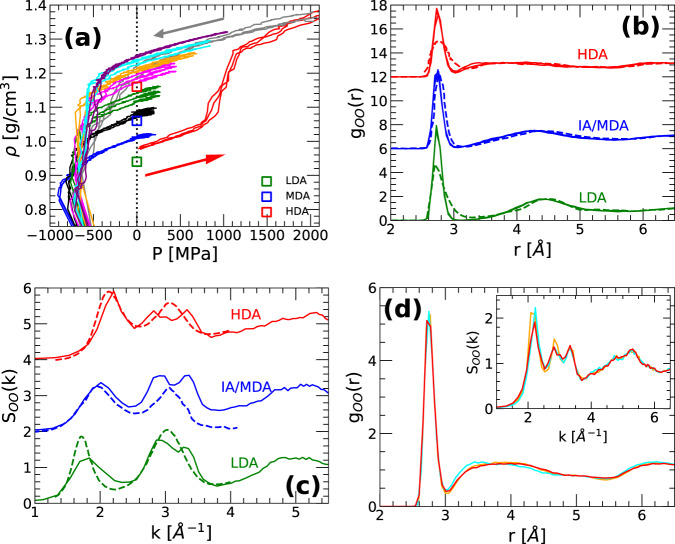
Thermodynamic and structural properties of amorphous ices during isothermal decompression at *T* = 80 K. **a** Density as a function of pressure during the isothermal decompression of the IA at *T* = 80 K. The IA are prepared by isobaric cooling at *P* = 100, 125, 200, 400, 600, 800 and 1000 MPa down to *T* = 80 K [cooling rate *q*_*T*_ = 10 K/ns]; they are then decompressed until fracture occurs at *P* < − 600 (approximately *ρ* < 0.85 g/cm^3^) [blue, black, green, magenta, orange, cyan, and violet (bottom-to-top)]. Three independent runs are shown for each decompression (decompression rate *q*_*P*_ = 100 MPa/ns). The (three) red lines are the *ρ*(*P*) during the compression-induced LDA-to-HDA transformation (*P* = 0.1 to 3000 MPa). The gray lines are the subsequent decompression of HDA (*P* = 2000 MPa to *P* < 0) [compression/decompression rate *q*_*P*_ = 100 MPa/ns]. Green, blue, and red open squares correspond to the experimental densities of LDA, MDA, and HDA at *P* = 0.1 MPa and *T* ≈ 80 K, respectively (from refs. 2,9,36). **b** Comparison of the OO radial distribution function and (**c**) structure factor of LDA, MDA, and HDA at *P* = 0.1 MPa and *T* = 80 K from experiments^36,50,51^ (dashed lines) and amorphous ices obtained from our computer simulations of q-TIP4P/F water (solid lines). The green solid line corresponds to LDA; the red solid line corresponds to the HDA produced by the compression-decompression cycle shown in (**a**). The blue solid line corresponds to the IA obtained by cooling at *P* = 125 MPa; this IA has a similar density and structure as the MDA made by ball-milling of ice *I*_*h*_ in ref. 36. **d** OO RDF and *S*(*k*) (inset) of the (i) IA obtained in the computer simulations by cooling at *P* = 600 − 800 MPa (orange and cyan lines in (**a**), respectively), and (ii) the HDA of q-TIP4P/F water obtained by isothermal compression/decompression starting from LDA (red and gray lines). Both amorphous ices exhibit remarkably similar OO RDF and *S*(*k*) at *P* = 0.1 MPa and *T* = 80 K; they also have very similar densities [see orange, cyan, and gray lines in (**a**) at *P* = 0.1 MPa].

We first compare the LDA, IA, and HDA at *P* = 0.1 MPa and *T* = 80 K. To do so, we take the IA obtained at the cooling pressures *P* = 0.1 − 1000 MPa and *T* = 80 K, and subject them to isothermal decompression (at *T* = 80 K) to negative pressures, until the IA fracture. The density of the IA forms during the decompression runs are shown in Fig. 3a. Also included is the density of the system during the compression-induced LDA-to-HDA transformation at *T* = 80 K and the subsequent decompression-induced HDA-to-LDA transformation (*T* = 80 K). The LDA-to-HDA transformation during compression corresponds to the sudden increase in density at *P* ≈ 1000 MPa. The HDA-to-LDA transformation is not reversible at *T* = 80 K and *P* > 0 MPa, consistent with experiments^1^. Indeed, HDA as well as the IA produced at high-pressures all seem to transform to an LDA-like state during decompression before they fracture at *P* < − 600 MPa (see, e.g., refs. 29,31 for a discussion of the common behavior of amorphous ices as found in computer simulations during the pressure-induced LDA-HDA transformations).

Next, we compare the OO RDF and *S*(*k*) of LDA, MDA, and HDA reported from experiments with the corresponding results obtained for our IA at different pressures. All amorphous ices are compared at *T* = 77 − 80 K and after decompression (recovery) to *P* = 1 bar. We stress that the structure (RDF, *S*(*k*), and IR spectra) of the IA are barely altered during decompression; see Supplementary Fig. S4 and Supplementary Note 4. The experimental OO RDF and *S*(*k*) of LDA, MDA, and HDA are reported in refs. 36,50,51, and are shown in Fig. 3b, c (solid lines). In agreement with previous computational studies^52,53^, the OO RDF of q-TIP4P/F water LDA (obtained by isobaric cooling at *P* = 0.1 MPa) and HDA (obtained during the compression/decompression LDA-HDA cycle at *T* = 80 K) are in relative good agreement with the experimental OO RDF of LDA and HDA (green and red solid/dashed lines). Similarly, the *S*(*k*) of q-TIP4P/F water for LDA and HDA are in relative good agreement with the experimental data [Fig. 3c]. In particular, the location of the first peak of *S*(*k*) for LDA and HDA are well-reproduced by our computer simulations. Interestingly, while the experimental data usually shows a wide second peak in *S*(*k*) at *k*_2_ ≈ 3.1 Å^−1^, our simulations show this peak being split into two small peaks (in the experiments of ref. 54, a split second peak in *S*(*k*) has been reported for the case of LDA at normal pressure). The small differences in the RDF and *S*(*k*) of LDA and HDA, relative to the experimental data, are probably related to the slightly different densities of LDA/HDA in experiments and q-TIP4P/F water. As shown in Fig. 3a, the densities of LDA and HDA in our simulations are ≈ 0.98 g/cm^3^ and ≈ 1.21 g/cm^3^ (red and gray lines at *P* = 0.1 MPa), respectively, which are slightly larger than the corresponding experimental densities, *ρ*_*LDA*_ = 0.94 g/cm^3^ and *ρ*_*HDA*_ = 1.17 g/cm^3^^2,9^ [green and red squares], i.e., *δρ* = + 0.04 g/cm^3^ in both cases, for the studied cooling/compression/decompression rates.

Similarly, as shown in Fig. 3b, c, the experimental OO RDF and *S*(*k*) of MDA^36^ is well reproduced by our IA obtained at *P* = 125 MPa. In addition, the density of the IA obtained at *P* = 125 MPa is ≈ 1.07 g/cm^3^ [black lines in Fig. 3a at *P* = 0.1 MPa] which is close to the reported density of MDA, *ρ*_*MDA*_ = 1.06 g/cm^3^ (blue square). We note that the cooling pressure *P* = 125 MPa at which IA resembles MDA is slightly smaller than the LLCP pressure *P*_*c*_ ≈ 162 MPa. IA that structurally resemble MDA can probably be produced in a range of pressures near *P*_*c*_ so, upon isobaric cooling, LDA- and HDA-like domains composed of a few molecules (see, e.g., Fig. 2b) can be frozen in during the cooling process; the cooling rate should also play an important role in the resulting IA (see ‘Summary and Discussion’). Our results suggest that the experimentally-observed MDA, which is produced by crushing ice *I*_*h*_ (ball-milling), is somehow related to the IA obtained by isobaric cooling under pressure. However, while this *may* be the case, it is not impossible that two amorphous ices with very different preparation/history can exhibit the same density and structure^55^. Accordingly, our findings do not necessarily imply that MDA is related to the liquid state of water, i.e., that MDA can be obtained from the liquid state by a specific thermodynamic path; see also section ‘Nature of LDA, HDA, and IA within the potential energy landscape formalism’.

Interestingly, Fig. 3d shows that, at *P* = 0.1 MPa and *T* = 80 K, the OO RDF and *S*(*k*) of the IA obtained by cooling at *P* = 600−800 MPa practically overlap with the OO RDF and *S*(*k*) of HDA [obtained during the compression/decompression cycle at *T* = 80 K, red and gray lines in Fig. 3a]. Similar results hold for the IR spectra. In addition, the densities of these two amorphous ices practically overlap; see the orange, magenta, and gray lines in Fig. 3a at *P* = 0.1 MPa. Therefore, at least from the thermodynamic (density, temperature, and pressure) and structural point of view (RDF, *S*(*k*), IR spectra), these amorphous ices are apparently identical. However, from a fundamental point of view, these amorphous ices, are indeed different (see section 'Nature of LDA, HDA, and IA within the potential energy landscape formalism' and ref. 55).

### Nature of LDA, HDA, and IA within the potential energy landscape formalism

In order to provide a further understanding of the nature of LDA, HDA, and the IA obtained at different pressures, we characterize these amorphous ices using the PEL formalism. Specifically, within the PEL formalism, a given glass configuration can be unequivocally identified with an IS of the PEL (the local minimum of the PEL basin where the glass resides). Therefore, two glasses are identical if they are associated to the same IS. It follows that, from a thermodynamic/statistical mechanics point of view, one may expect that two macroscopic glass samples obtained by following different preparation protocols may be considered to be in the same glass ‘state’ if the average topographic properties of the PEL (depth, curvature, etc) associated to each glass sample are identical. Indeed, in a previous study^55^, we found that amorphous ices that share the same values of $({E}_{IS},\,{P}_{IS},\,{{{{{{{\mathcal{S}}}}}}}})$ and (*N*, *V*, *T*) exhibit practically identical behaviors when subjected to isothermal compression and heating, suggesting that these six PEL/thermodynamic quantities play the role of state variables for glasses. By the same reasoning, glasses with different values of $({E}_{IS},\,{P}_{IS},{{{{{{{\mathcal{S}}}}}}}})$, but with same values of (*N*, *V*, *T*), are expected to be in different glass ‘states’ since they necessarily reside in different regions of the PEL. For example, computer simulations show that the regions of the PEL associated to the LDA and HDA *families* of amorphous ices are characterized by different values of *E*_*IS*_, *P*_*IS*_, and/or ${{{{{{{\mathcal{S}}}}}}}}$. Interestingly, computer simulations show an apparent potential energy barrier or a concavity in *E*_*IS*_(*V*) when LDA-HDA are interconverted by isothermal compression-decompression^44,56^; for equilibrium systems, the presence of a concavity in *E*_*IS*_(*V*) is consistent with the presence of a first-order phase transition at low temperatures^56^.

#### Isobaric cooling of water and the IA

**Fig. 4:**
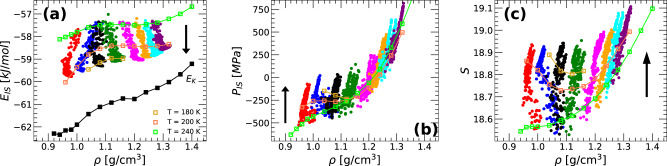
Inherent structure properties of liquid water during vitrification (isobaric cooling) at different pressures. Average (**a**) IS energy, (**b**) IS pressure, and (**c**) basin shape function of q-TIP4P/F water during isobaric cooling, from *T* = 240 K (liquid) to *T* = 80 K (amorphous ice), at pressures *P* = 0.1, 100, 125, 200, 400, 600, 800 and 1000 MPa (left-to-right: red, blue, black, green, magenta, orange, cyan, and violet, respectively); cooling rate *q*_*T*_ = 10 K/ns. For comparison, also included are the values of *E*_*IS*_(*ρ*), *P*_*IS*_(*ρ*), and ${{{{{{{\mathcal{S}}}}}}}}(\rho )$ for liquid water in equilibrium at *T* = 180, 200, 240 K (open squares). Upon cooling (see arrows), q-TIP4P/F water explores increasingly deeper regions of the PEL with the corresponding basins becoming slightly thinner as *T* decreases (larger curvature and ${{{{{{{\mathcal{S}}}}}}}}$); *P*_*IS*_(*T*) also increases upon cooling.

**Fig. 5:**
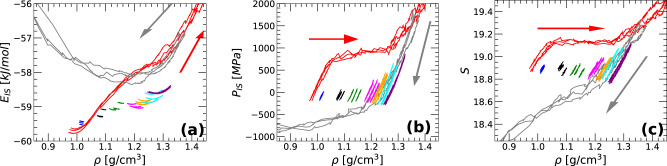
Inherent structure properties of amorphous ices during isothermal decompression at *T* = 80 K. Average (**a**) IS energy, (**b**) IS pressure, and (**c**) basin shape function of the IA during isothermal decompression at *T* = 80 K [see Fig. 3a]. The IA are formed by isobaric cooling at different pressures *P* down to *T* = 80 K. The resulting IA are then decompressed from the corresponding cooling pressure down to *P* = 0.1 MPa (*T* = 80 K); *P* = 100, 125, 200, 400, 600, 800, and 1000 MPa correspond to blue, black, green, magenta, orange, cyan, and violet, respectively. Also included are the *E*_*IS*_, *P*_*IS*_, and ${{{{{{{\mathcal{S}}}}}}}}$ of the amorphous ices accessed by the system during the compression-induced LDA-to-HDA transition (red lines) and subsequent decompression of HDA (gray lines). The different values of *E*_*IS*_, *P*_*IS*_, and/or ${{{{{{{\mathcal{S}}}}}}}}$ associate to the IA and amorphous ices accessed during the LDA-to-HDA transformation (red lines) imply that these are different amorphous ices. The IA are located in deeper regions of the PEL (lower *E*_*IS*_) relative to regions of the PEL accessed by water during the compression-induced LDA-to-HDA transformation (red lines), suggesting that isobaric cooling (IA, rate *q*_*T*_ = 10 K/ns) produces more stable amorphous ices than isothermal compression of LDA (*q*_*P*_ = 100 MPa/ns); see text.

Figure 4a shows *E*_*IS*_(*ρ*) during the preparation of the IA, i.e., during the isobaric cooling runs included in Fig. 1a. Note that, for clarity, only the temperatures in the range *T* = 240 − 80 K are included in Fig. 4a while all temperatures *T* = 240 − 0 K are shown in Fig. 1a. Upon cooling, *E*_*IS*_ decreases monotonically implying that the system explores increasingly deeper regions of the PEL. At *T* = 80 K, the IA samples IS with energies comparable to those explored by equilibrium q-TIP4P/F water at *T* = 180 − 200 K, depending on the pressure. These IS are located in regions of the PEL that are relatively higher than those regions associated to the predicted ideal glass. Specifically, the IS energies of the IA at *T* = 80 K are ≈ 1.5 − 2.0 kJ/mol larger than the corresponding IS energy of the amorphous ices at the Kauzmann temperature, *E*_*IS*_(*T*_*K*_) (black line and squares)^40^ [as expected, the IS energy of LDA and HDA are also higher than *E*_*IS*_(*T*_*K*_); see Fig. 5a]. As shown in ref. 57, reducing the cooling rate allows the system to reach even deeper IS within the PEL but *E*_*IS*_(*T*_*K*_) marks the lowest IS energy accessible to the glass state.

Figure 4a also shows that, depending on the cooling pressure, the system moves on the PEL towards different regions (of the PEL). Specifically, upon cooling, *E*_*IS*_(*ρ*) has a positive slope at *P* ≤ 100 MPa, and a negative slope at *P* ≥ 200 MPa; the case *P* = 125 MPa is less evident with the density remaining approximately constant upon cooling. This is, of course, due to the evolution of *ρ*(*T*) in Fig. 1a. Yet, from the PEL perspective, our results provide a nice and intuitive understanding of how the system is evolving upon cooling and why that is the case. As shown in previous works^56^, computer simulations of amorphous ice suggest that there are two well-separated regions in the PEL of water. For example, pressure-induced LDA-HDA transformations in ST2 water show that the system travels between two megabasins of the PEL upon compression/decompression, one region is associated to LDA and the other to HDA. Moreover, the LDA/HDA megabasins are separated by an apparent energy barrier^56^. Similar results hold for TIP4P/2005 water although, in this case, the LDA and HDA regions of the PEL are apparently separated by a region where *E*_*IS*_(*V*) is concave, i.e., ${\left(\partial {E}_{IS}/\partial V\right)}_{N,T} < 0$ (with no evidence of an energy barrier). Our results for q-TIP4P/F water are consistent with those for TIP4P/2005 water^44^. Specifically, in Fig. 4a, the IS energy of the ideal amorphous ices *E*_*IS*_(*T*_*K*_) shows a concave region in the range ≈ 0.97−1.20 g/cm^3^. Therefore, our computer simulations supports the view where, upon isobaric cooling, the system (water) travels downwards on the PEL towards (a) the LDA region of the PEL at *P* ≤ 100 MPa, and (b) the HDA region of the PEL at *P* ≥ 200 MPa; (c) at *P* = 125 MPa, the system evolves towards IS located in the intermediate region of the PEL, in between the regions corresponding to LDA and HDA.

Figure 4b, c show the evolution of *P*_*IS*_ and ${{{{{{{\mathcal{S}}}}}}}}$ upon isobaric cooling, during the preparation of the IA. Upon cooling, both *P*_*IS*_ and ${{{{{{{\mathcal{S}}}}}}}}$ increase monotonically at all pressures studied. At the lowest *T* included in Fig. 4b (*T* = 80 K), *P*_*IS*_ < 0 for *ρ* < 1.0 g/cm^3^ indicating that these IA remain under tension as *T* → 0; *P*_*IS*_ > 0 for *ρ* < 1.0 g/cm^3^. The slight increase of ${{{{{{{\mathcal{S}}}}}}}}$ upon cooling [Fig. 4c] implies that the curvature of the basins sampled by water (about the corresponding IS) increases as well. In other words, as the *T* decreases, liquid water/IA sample ‘thinner’ basins. Our results are consistent with previous PEL studies of ST2^56,57^ and TIP4P/2005^44^ water that focused on the pressure-induced LDA-HDA transformations.

#### IA, LDA, and HDA

Next, we compare the IA, LDA, and HDA of q-TIP4P/F water at *T* = 80 K and *P* = 0.1 MPa using the PEL approach. To do so, we calculate the PEL properties of these amorphous ices during the decompression runs included in Fig. 3a. However, for clarity, Fig. 5 only includes pressures down to *P* = 0.1 MPa while Fig. 3 shows results also for negative pressures. During decompression, the IA exhibit mild changes in *E*_*IS*_(*ρ*) and ${{{{{{{\mathcal{S}}}}}}}}$ implying that the IA remain at practically the same depth within the PEL, and that the curvature of the corresponding IS barely changes (decreases) upon decompression (*T* = 80 K). In addition, *P*_*IS*_(*ρ*) also decreases during decompression, particularly in the case of IA produced at high pressures. At *ρ* = 1.0 g/cm^3^, *P*_*IS*_ < 0 for all the IA studied. We summarize the main findings below.

(i) One of the most important points of Fig. 5 is that, at the cooling and compression/decompression rates studied, the IS sampled by the IA during the decompression runs (*T* = 80 K) are all different than the IS sampled by the system during the pressure-induced LDA-HDA transformation cycle at *T* = 80 K. In other words, the values of *E*_*IS*_, *P*_*IS*_, and/or ${{{{{{{\mathcal{S}}}}}}}}$ of the amorphous ices at *P* = 0.1 MPa and *T* = 80 K depend on whether the amorphous ice is prepared by cooling or by compression/decompression. Importantly, this conclusion also applies to the IA obtained at *P* = 600 − 800 MPa. While the IA prepared at *P* = 600 − 800 MPa have practically the same density and structure as HDA at *P* = 0.1 MPa and *T* = 80 K [Fig. 3a, d], as well as same *P*_*IS*_ and ${{{{{{{\mathcal{S}}}}}}}}$ [Fig. 5b, c], these IA and HDA forms have different values of *E*_*IS*_ [orange, magenta, and gray lines in Fig. 5a]. Briefly, the IA obtained at *P* = 600 − 800 MPa and rate *q*_*T*_ = 10 K/ns, and the HDA produced by the compression of LDA (*q*_*P*_ = 100 MPa/ns) are different amorphous ices.
(ii) It is particularly interesting that the *E*_*IS*_(*ρ*) of the IA are all lower than the *E*_*IS*_(*ρ*) of the amorphous ices produced during the compression-induced LDA-to-HDA transformation (red lines). In other words, by performing isobaric cooling (*q*_*T*_ = 10 K/ns), it is possible to reach deeper regions of the PEL that are not accessible during the compression of LDA (*q*_*P*_ = 100 MPa/ns). Computer simulations show that, for amorphous ices at a given density or pressure, the lower the value of *E*_*IS*_, the more stable the corresponding glasses are (for example, *E*_*IS*_ decreases monotonically during annealing hyperquenched glassy water^57^). It follows that, for the rates considered here, the IA are always more ‘stable’ than the amorphous ices sampled during the compression of LDA.

We stress again that our conclusions are based on the rates *q*_*T*_ = 10 K/ns and *q*_*P*_ = 100 MPa. These cooling/compression/decompression rates were chosen so the total simulation time of a cooling run is of the same order of magnitude as the total simulation time of the compression-induced LDA-to-HDA and decompression-induced HDA-to-LDA transformations ( ≈20−30 ns in all cases). However, the values of *q*_*T*_ and *q*_*P*_ are important in computational studies as well as experiments that access <1*μ*s time scales^18,20,21,27,28^. For example, as shown in ref. 44 for the case of TIP4P/2005 water, reducing *q*_*T*_ from 30 to 0.01 K/ns decreases the IS energy of LDA (or, equivalently, the IA formed at *P* = 0.1 MPa) by ≈ 1 kJ/mol (the rate 0.01 K/ns is approximately the *slowest* cooling rate that can be used in experiments to avoid crystallization^32,33^). Similarly, reducing *q*_*P*_ from 300 to 0.1 MPa/ns decreases the IS energy of amorphous by ≈1 kJ/mol (at *ρ* > 1.1 g/cm^3^). We note that reducing the compression/decompression rate from *q*_*P*_ = 100 MPa/ns to *q*_*P*_ = 10 MPa/ns does *not* affect our results; see Supplementary Fig. S5 and Supplementary Note 5. Nonetheless, it may be possible that particular combinations of *q*_*T*_ and *q*_*P*_ may lead to IA with values of *E*_*IS*_ that are also comparable, or even larger, than the *E*_*IS*_ of the amorphous ices produced upon isothermal compression of LDA (including HDA). If so, it may be possible to produce the same amorphous ice by (a) isothermal compression of LDA, and (b) isobaric cooling of liquid water under pressure.

## Summary and discussion

We performed MD simulations of amorphous ices prepared by different thermodynamic paths, (i) isobaric cooling of liquid water at *P* = 0.1−1000 MPa, and (ii) isothermal compression/decompression at *T* = 80 K. Process (ii) leads to the well-known pressure-induced interconversion of LDA and HDA. Interestingly, and consistent with previous studies^42,43,45^, we find that process (i) can produce a continuum of amorphous ices (IA) with densities in-between the densities of LDA and HDA (*ρ*_*LDA*_ = 0.94 g/cm^3^ and *ρ*_*HDA*_ = 1.15−1.17 g/cm^3^^2,9^). In particular, it was shown that the IA produced by isobaric cooling at *P* ≈ 125 MPa has a remarkably similar density and structure (OO radial distribution function and structure factor) of the recently discovered MDA^36^. We note that MDA is made by crushing (ball-milling) ice *I*_*h*_ while IA is produced by vitrifying liquid water. While our results do not conclusively imply that MDA is related to the liquid state of water, they do show that MDA is not unique (i.e., an infinite number of medium-density amorphous ices may exist), and that MDA-like amorphous ices can indeed be generated by cooling under pressure (with the same density and remarkably similar structures as MDA).

Amorphous ices produced at *P* < 1000 MPa have been usually classified in the past as belonging to the LDA and HDA *family*. This is because, experimentally, such amorphous ices have densities around *ρ*_*LDA*_ = 0.94 g/cm^3^ or *ρ*_*HDA*_ = 1.15−1.17 g/cm^3^ when recovered at *T* = 77 K and *P* = 0.1 MPa. Accordingly, the discovery of MDA and, more generally, IA may be surprising and one may wonder, what is the nature of these amorphous ices? To address this question, we used the potential energy landscape formalism. Our results are consistent with a PEL for water that has two well-separated regions, one associated to LDA and another to HDA, and where the IA produced at *P* ≈ 100 − 200 MPa correspond to IS that are in the intermediate region of the PEL, between the regions associated to LDA and HDA. The PEL formalism also provides a nice and intuitive understanding of the evolution of liquid water upon cooling, during its vitrification into IA. Specifically, upon cooling at approximately *P* < 125 MPa (*P* > 125 MPa), the system moves downwards on the PEL towards the LDA (HDA) regions of the PEL; at *P* ≈ 125 MPa, the system moves downwards the PEL until it becomes trapped in the transition region of the PEL.

**Fig. 6:**
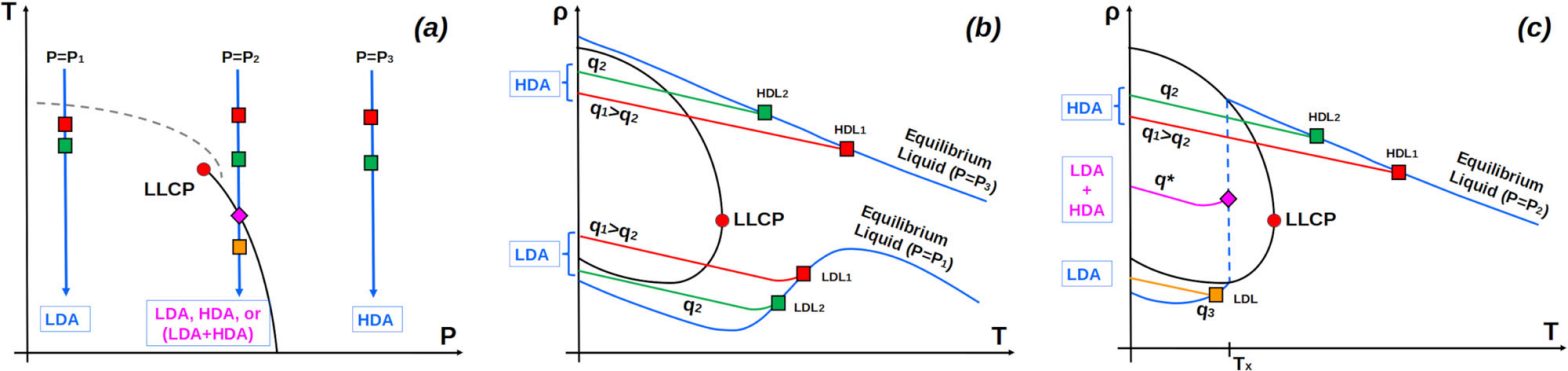
Schematic diagrams showing the effects of varying the cooling rate on the nature of the resulting IA, at different pressures. **a** Thermodynamic paths in the *P* − *T* plane (blue lines) that can be used for the vitrification of liquid water by isobaric cooling at three different pressures: (i) *P* < *P*_*c*_, (ii) *P* > *P*_*c*_, crossing the LLPT (black) line, and (iii) *P* > *P*_*c*_, avoiding the LLPT line. Isobaric cooling along thermodynamic paths (i) and (iii) produces LDA and HDA, respectively; isobaric cooling along path (ii) can produce LDA, HDA, or a mixture of LDA and HDA, depending on the cooling rate (see text). The red circle represents the LLCP; the dashed gray line corresponds to the density maxima line. **b** Density of water as a function of temperature during isobaric cooling along the paths (i) and (iii) shown in (**a**). The blue lines represent the density of water in the equilibrium liquid state. The red and green lines indicate the out-of-equilibrium paths followed by the liquid during vitrification (isobaric cooling) using a slower (*q*_2_; green line) and faster cooling rate (*q*_1_ < *q*_2_; red line). Squares indicate the state at which the system departs from equilibrium. For comparison, we also show the binodal line (solid black line) enclosing the LL coexistence region, and the LLCP (red circle). Along path (i) [path (iii)], liquid water vitrifies into an LDA (HDA) form. **c** Same as (**b**) for path (ii) shown in (**a**). Depending on the cooling rate, liquid water may produce HDA [at rates *q*_1_ and *q*_2_ (*q*_1_ > *q*_2_); green and red lines], a phase-separated LDA+HDA amorphous ice [rate q^*^ (*q*_2_ > q^*^ > *q*_3_); magenta line], and LDA (rate *q*_3_; orange line).

The q-TIP4P/F water model exhibits a LLCP and LLPT at low temperature. Hence, our results imply that the presence of amorphous ices with intermediate densities (IA) are not incompatible with the LLPT scenario. Indeed, supercritical amorphous ices obtained by vitrifying water under pressure at *T* > *T*_*c*_, at hyperquenching rates *q*_*T*_ > 10 K/ns, must be structurally similar to their parent (supercritical) liquid and hence, such amorphous ices must be homogeneous. In our isobaric cooling MD simulations that lead to IA, liquid water looses equilibrium at *T* ≈ 200−210 K (*q*_*T*_ = 10 K/ns), i.e., at temperatures slightly above the LLCP temperature *T*_*c*_ ≈ 197 K of q-TIP4P/F water, and enters the glass state at temperatures slightly below *T*_*c*_. Hence, it is not surprising that the resulting IA are homogeneous, with no ‘phase-separation’ into LDA and HDA. Much slower cooling rates, accessible (inaccessible) to experiments (simulations), may allow liquid water to phase-separate into low-density and high-density liquid water before the system is vitrified^18,21^ (if crystallization can be avoided). At such (low) rates, it may be possible to make amorphous ices with intermediate densities between those of LDA and HDA, however such amorphous ices would exhibit phase separation of LDA and HDA. Evidently, such amorphous ices would not correspond to an IA. To clarify this point, we include in Fig. 6a the thermodynamic path in the P-T plane followed during the vitrification of liquid water by isobaric quenching at three different pressures. Path 1, at *P* < *P*_*c*_, corresponds to the vitrification of water into LDA (as done in the experiments by Mayer at *P* = 1 bar^32^); path 3, at *P* > *P*_*c*_, corresponds to the vitrification of water into HDA (as done in the experiments by Suzuki and Mishima at *P* ≈ 300 MPa^34^). Path 2 is at a pressure slightly above *P*_*c*_ and such that it crosses the equilibrium LLPT line (in this discussion, we assume that crystallization is avoided at the cooling rates considered). Figure 6b shows the density of the equilibrium liquid along paths 1 and 3 (blue lines), together with the binodal lines and LLCP in the *ρ* − *T* plane. For each path, we also include two out-of-equilibrium trajectories followed by the system during isobaric vitrification at different cooling rates (red and green lines). Figure 6c is analogous to Fig. 6b for path 2 which enters the LL coexistence region. *Path 1:* At *P* < *P*_*c*_, the equilibrium liquid never enters the coexistence region in the *ρ* − *T* plane. Due to the existence of a density maxima, the density of the equilibrium liquid decreases rapidly upon cooling (potentially, reaching a density minimum), and remains below the coexistence region at very low temperatures. The red and green lines correspond to the vitrification of the liquid using a slower (*q*_2_) and a faster (*q*_1_ > *q*_2_) cooling rate, respectively. The corresponding thermodynamic states at which the system departs from equilibrium are indicated by LDL1 and LDL2. The system vitrifies at lower temperatures below which *ρ* increases linearly with T upon further cooling (note that nuclear quantum effects can weaken the *T*-dependence of the density in amorphous ices^41^). At this low pressure, depending on the cooling rate, the final amorphous ice formed at *T* = 80 K may (*q*_1_) or may not (*q*_2_) be located within the coexistence region. In practice, due to the excessively fast rates (>10^6^ K/s) needed to avoid crystallization at *P* ≈ 1 bar, the final amorphous ice (LDA) most probably is located within the coexistence region *ρ* - *T* plane. *Path 3:* A similar situation holds at *P* > *P*_*c*_, where the LLPT line is not crossed upon cooling [Fig. 6a]. As shown in Fig. 6b, the liquid departs from equilibrium at different thermodynamic states (HDL1 and HDL2) depending on the cooling rate. At the pressure considered, both amorphous ices (HDA) produced at *T* = 80 K are located within the coexistence region in the *ρ* − *T* plane. Interestingly, while the density of LDA decreases with decreasing cooling rates [path 1], the density of HDA increases with decreasing cooling rates (path 3). *Path 2:* The case of path 2 is rather complex. As shown in Fig. 6c many outcomes are possible (depending on the cooling rate) at a pressure where the equilibrium liquid can cross the LLPT line [Fig. 6a]. At a very fast cooling rate (*q*_1_, *q*_2_), the system departs from equilibrium before entering the coexistence region [states HDL1 and HDL2], leading to amorphous ice at *T* = 80 K (HDA-like) located within the coexistence region in the *ρ* − *T* plane. At a very slow cooling rate (*q*_3_), the system may depart from equilibrium after exiting the coexistence region [states LDL], leading to amorphous ices at *T* = 80 K (LDA-like) located inside or outside the coexistence region in the *ρ* − *T* plane. As for paths 1 and 3, the density of these amorphous ices varies with the cooling rate. It may also be possible that there are intermediate cooling rates (q^*^ where *q*_2_ > q^*^ > *q*_3_) for which the system is vitrified while it is transforming from the HDL to LDL states, during the cooling process. While in equilibrium, liquid water can be found only in the HDL state at *T* > *T*_*x*_ and LDL states at *T* < *T*_*x*_ [see solid blue lines in Fig. 6c], during the cooling process, the system may be found in an out-of-equilibrium state visited during the transformation from HDL to LDL [dashed line in Fig. 6c]. The magenta diamond in Fig. 6c represents one of such an out-of-equilibrium states at which the liquid is phase-separated with LDL and HDL domains. The magenta line in Fig. 6c represents the case where the system is vitrified upon cooling at rate q^*^ from the phase-separated liquid state (diamond). The resulting glass is expected to be composed of LDA and HDA domains separated by sharp glass-glass interfaces. Hence, such glasses would not be homogeneous and hence, they would be fundamentally different from MDA and the IA found in our MD simulations.

The results of this work should be helpful for our understanding of the nature of amorphous ices, including MDA. In particular, we note that the IA may play a relevant role in important technological applications, such as cryo-electron microscopy (cryoEM)^58–60^, cryopreservation techniques^61–64^, and x-ray experiments of protein crystals^65,66^. While vitrification of water under pressure is not uncommon in cryoEM experiments, experimental/computational studies characterizing the properties of amorphous ices generated by isobaric cooling under pressure (i.e., IA) are rather limited^34,42,43,45,64,67^. Indeed, most experimental studies exploring the phase behavior of amorphous ice are based on LDA and HDA forms that are produced by isothermal compression/decompression of ice *I*_*h*_, LDA, and HDA; vitrification of water has been mostly limited to *P* = 0.1 MPa^32,33^.

Our work also raises the question of whether intermediate amorphous ices, analogous to the IA produced here by isobaric cooling under pressure, can also be formed via other thermodynamic paths. For example, a few experiments^46,68–70^ have identified intermediate amorphous ice structures produced by heating specific forms of HDA at *P* = 1 bar, during the transformation of HDA to LDA. Similar intermediate amorphous structures may be accessible via isobaric heating of LDA under pressure, or along any thermodynamic path that does not intersect the LLPT line in the P-T plane and where LDA/HDA must transform to HDA/LDA (e.g., along a path where the system crosses the spinodal lines associated to the LLPT, extended into the glass domain). The nature of these amorphous ices, and how they compare with our IA (and MDA) remains unclear.

## Methods

We perform out-of-equilibrium molecular dynamics (MD) simulations of a system composed of *N* = 512 water molecules in a cubic box with periodic boundary conditions [increasing the system size to *N* = 5118 does not affect our results; see Supplementary Note 1 and Supplementary Fig. S1]. Water molecules are represented using the q-TIP4P/F model^71^. This is a flexible model based on the rigid TIP4P/2005 water model^72^, which has been used extensively in previous MD simulations to study liquid water, aqueous solutions, ice, and glassy water^24,44,73–76^. The q-TIP4P/F water model incorporates intramolecular flexibility by modeling the O-H covalent bond potential energy with a fourth-order polynomial expansion of a Morse potential and a simple harmonic potential to model the potential energy of the HOH angle. The q-TIP4P/F water model has been optimized to be used in path-integral molecular dynamics (PIMD) simulations and it reproduces remarkably well the properties of liquid water^71,77,78^ as well as ice *I*_*h*_ and LDA at *P* = 0.1 MPa^41,79^. At low pressures and approximately *T* > 150 K, differences in many of the thermodynamic (e.g., density, isothermal compressibility), structural (e.g., radial distribution functions), and dynamic properties (e.g., diffusion coefficient) of liquid water obtained from PIMD simulations and classical MD simulations are minor, if any^41,77^. This is due to the approximate cancellation of competing quantum effects in q-TIP4P/F water^71^. Differences between classical MD and PIMD simulations are observable in q-TIP4P/F liquid water at intermediate pressures and very low temperatures (*P* = 100 − 200 MPa and *T* < 230 K)^78^, as well as in the ice *I*_*h*_ and LDA states (*P* = 0.1 MPa and *T* < 150 K)^41,80,81^. However, even under such conditions, the phase behavior of ice/amorphous ice in q-TIP4P/F water reported from classical MD and PIMD (quantum) simulations is qualitatively similar to one another and, in particular, they are consistent with experiments^41,77,78^.

We perform two kinds of out-of-equilibrium MD simulations, (i) isobaric cooling runs at different pressures, and (ii) isothermal compression/decompression runs at *T* = 80 K. In order to improve statistics, three independent runs are performed for each case. (i) Isobaric cooling runs are performed at *P* = −100, 0.1, 50, 100, 115, 125, 140, 150, 160, 175, 190, 200, 300, 400, 500, 600, 700, 800, 900, and 1000 MPa to produce amorphous ice. During these MD simulations, an equilibrium liquid configuration obtained at *T* = 240 K (and at a given *P*) is cooled down to *T* = 0 K using a constant cooling rate. The isobaric cooling runs at *P* = 0.1 MPa produce LDA; similarly, as we will show below, isobaric cooling of liquid water at high pressures (e.g., *P* > 500 MPa) produces HDA-like states. During the cooling simulations, the thermostat temperature is reduced linearly with time, at a rate *q*_*T*_ = 10 K/ns. At this cooling rate, the total simulation time to go from 240 K to 80 K is 16 ns and hence, the liquid loses equilibrium before its *α*-relaxation time becomes of the order 10–15 ns. The effect of reducing *q*_*T*_ in low-pressure amorphous ices has been explored in detail in ref. 44. The employed cooling rate is ≈ 3 orders of magnitude faster than the experimental rate ≈ 0.01 K/ns^32,33^ used to vitrify water at *P* = 0.1 MPa – experiments employing a slower cooling rate lead to ice formation. Nonetheless, the LDA states obtained in computer simulations with rate *q*_*T*_ = 10 K/ns are qualitatively similar to those obtained in experiments^52^.

(ii) The amorphous ices obtained by isobaric cooling in (i) at *T* = 80 K and pressure *P*, are then decompressed at constant temperature (*T* = 80 K) down to negative pressures until they fracture. During these isothermal decompression runs, the barostat pressure is decreased linearly with time, with a decompression rate of *q*_*P*_ = 100 MPa/ns. In addition, we also generate HDA by isothermal compression of LDA at *T* = 80 K. The starting LDA configurations are obtained as explained in (i), by cooling liquid water at *P* = 0.1 MPa down to *T* = 80 K. As shown in Fig. 3a, during the compression run (*q*_*P*_ = 100 MPa/ns), LDA suddenly transforms to HDA at *P* ≈ 1000 MPa. The resulting HDA formed at *P* = 2000 MPa is then decompressed at constant temperature (*T* = 80 K, *q*_*P*_ = 100 MPa/ns) until the amorphous ice fractures at very negative pressures.

All of our MD simulations are performed using the OpenMM software package (version 7.4.0)^82^. The temperature is controlled using the stochastic (local) path-integral Langevin equation (PILE) thermostat^83^ and a Monte Carlo (MC) barostat is used to maintain the pressure of the system^84^. To control the temperature, the thermostat collision frequency parameter is set to *γ* = 0.1 ps^−1^ during the isobaric cooling and isothermal compression/decompression runs. The frequency of the MC barostat is set to 25 simulation steps during the isobaric cooling and isothermal compression/decompression runs. In our MD simulations, the time step *dt* is set to 0.50 fs. Short-range (Lennard-Jones pair potential) interactions are calculated using a cutoff *r*_*c*_ = 1.0 nm and the long-range electrostatic interactions are computed using the reaction field technique^85^ with the same cutoff *r*_*c*_. In the reaction field technique, the dielectric constant (relative permittivity) of the continuum medium beyond the cutoff radius *r*_*c*_ is set to 78.3. The expression for the reaction field equation implemented in the OpenMM software package to model electrostatic interactions is given in Eq. 18.6.3 of ref. 82.

We also calculate relevant PEL properties of the system throughout the thermodynamic paths outlined in (i) and (ii). Briefly, the PEL of a system of *N* q-TIP4P/F water molecules is the hypersurface in (9*N* + 1)-dimensional space defined by the potential energy of the system *V*({*r*_*i*,*j*,*k*_}) as function of the 9*N* atom coordinates *r*_*i*,*j*,*k*_ (here, *i* = 1, 2. . *N* is the molecule number, *j* = 1, 2, 3 is the atom number of water molecule *i*, and *k* = *x*, *y*, *z*). Within the PEL formalism, the properties of the system are directly related to the PEL local minima (inherent structure, IS) that are visited by the system at a given (*N*, *V*, *T*). Accordingly, during the isobaric cooling and isothermal/compression/decompression runs, we save configurations of the system every 2.5 K (isobaric cooling) and 5 MPa (compression/decompression). For each of these configurations, we calculate the corresponding potential energy minimum (IS) using the L-BFGS-B algorithm^86^. The IS energy *E*_*IS*_ and pressure *P*_*IS*_ are obtained directly from the IS. For example, *P*_*IS*_ is the pressure given by the pressure virial expression at the IS. To quantify the curvature of the PEL basins about the IS sampled by the system, we also calculate the Hessian matrix of the system at the IS. The basins curvature is quantified by the shape function defined as,

1 $${{{{{{{\mathcal{S}}}}}}}}={\left\langle \mathop{\sum }\limits_{i = 1}^{9N-3}\ln \left(\frac{\hslash {\omega }_{i}({e}_{IS},V)}{{A }_{0}}\right)\right\rangle }_{{e}_{IS}}$$

In this expression, ${\{{\omega }_{i}^{2}\}}_{i = 1,2...9N-3}$ are the eigenvalues of the Hessian matrix^39,40^, *ℏ* is Planck’s constant in its reduced form, and A_0_ ≡ 1 kJ/mol is a constant that ensures that the arguments of the logarithm has no units.

### Supplementary information

Supplementary Material

## Data Availability

The authors confirm that the data supporting the findings of this study are available within the article and its supplementary material. The SM includes additional results from MD simulations where we explore the effects of system size and compression/decompression rates on the structural and thermodynamic properties of LDA, IA, and HDA.
