# Supplementary material for: A continuum of amorphous ices between low-density and high-density amorphous ice
Source: Commun Chem. 2024 Feb 20;7:36. doi: 10.1038/s42004-024-01117-2 (PMC10879119; doi:10.1038/s42004-024-01117-2)
Supplement: Supplementary file 1 — Supplementary Material [file 42004_2024_1117_MOESM1_ESM.pdf]

Supplementary Material for "A Continuum of Amorphous Ices between Low-Density and High-Density Amorphous Ice"

Ali Eltareb[1,2,*], Gustavo E. Lopez[3,4,*], and Nicolas Giovambattista[1,2,4*]

[1]*Department of Physics, Brooklyn College of the City University of New York, Brooklyn, New York 11210, United States*

[2]*Ph.D. Program in Physics, The Graduate Center of the City University of New York, New York, NY 10016, United States*

[3]*Department of Chemistry, Lehman College of the City University of New York, Bronx, New York 10468, United States*

[4]*Ph.D. Program in Chemistry, The Graduate Center of the City University of New York, New York, NY 10016, United States*

[*] ali.eltareb@brooklyn.cuny.edu, gustavo.lopez1@lehman.cuny.edu, ngiovambattista@brooklyn.cuny.edu

I. SUPPLEMENTARY NOTE 1

A. System Size Effects

We confirm that our results in the main manuscript are robust relative to the system size. To do so, we compare in Fig. S1 a few relevant properties of the amorphous ices shown in the main manuscript, based on a system composed of $N = 512$ molecules, with results from MD simulations of an $N = 5118$-molecule system. We employ the same techniques and parameters used in the main manuscript, including the cooling/compression/decompression rates. The system size effects in Fig. S1 are negligible.

II. SUPPLEMENTARY NOTE 2

A. Structure Factor

In the main manuscript, we compare the structure factor $S(k)$ of the intermediate amorphous (IA) ices obtained by cooling at different pressures. Here, we focus on the location of the first-peak of $S(k)$. Fig. S2 shows the location of the structure factor first-peak, $k_1(P)$, of the equilibrium liquid at $T = 240$ K (magenta and green symbols) and the corresponding IA at $T = 80$ K (brown and blue symbols), as a function of pressure P and density ρ. $k_1(P)$ shown in Fig. S2(a) increases with increasing pressure, with a pronounced but rather continuous increase as the IA evolve from LDA-like, at low cooling pressure, to HDA-like at higher pressures. Similarly, $k_1(\rho)$ [Fig. S2(b)] is a continuous function of the density, which indicates that $k_1(P)$ exhibits no discontinuity for the IA states (since $\rho(P)$ is also a continuous function of P).

III. SUPPLEMENTARY NOTE 3

A. IR Spectra

Figs. S3(a) and S3(b) show magnifications of the IR spectra for the IA included in Fig. 1(h), for the frequency ranges (i) $\omega < 1800$ cm^{-1} and (ii) $\omega > 3000$ cm^{-1}. The changes in the IR spectra with increasing cooling pressures are rather mild and are not inconsistent

with experiments. For comparison, we show in Fig. S3(c) the IR spectra of the IA in the OH stretching region [$\omega > 3000$ cm^{-1}] together with the corresponding experimental data for LDA and HDA reported in Refs. [2, 3]. The experimental IR spectra are shifted by $\delta\omega = +200$ cm^{-1}. Nonetheless, in both cases, we see that the IR maximum in Fig. S3(c) shifts towards large frequencies as the system evolves from LDA to HDA.

IV. SUPPLEMENTARY NOTE 4

A. Structural Changes in the IA During Decompression

Here, we compare the structure of the IA produced at $T = 80$ K (i) after isobaric cooling at pressure $P > 0$, and (ii) after further isothermal decompression from the corresponding cooling P down to $P = 0.1$ MPa (at $T = 80$ K). Fig. S4 shows the OO radial distribution function, structure factor $S(k)$, and infrared spectra of the IA at $T = 80$ K and prepared at $P = 100,\ 400,\ 1000$ MPa. The studied structural properties of the IA before (solid lines) and after the isothermal decompression (dashed-lines) are barely affected.

V. SUPPLEMENTARY NOTE 5

A. Compression rate dependence: $q_P = 10, 100$ MPa/ns

In this section, we study the effects of varying the compression/decompression rate, q_P, on the PEL properties sampled by the system during the pressure-induced LDA-HDA transformations at $T = 80$ K. Specifically, we compare the evolution of the IS energy, IS pressure, and shape function (E_{IS}, P_{IS}, and $\mathcal{S}$) during the LDA-HDA transformation with compression/decompression rates $q_P = 10,\ 100$ MPa/ns. As shown in Fig. S5, the values of $E_{IS}(\rho)$, $P_{IS}(\rho)$, and $\mathcal{S}(\rho)$ during the pressure-induced LDA-HDA transformation with compression

rates $q_P = 10,\ 100$ MPa/ns practically overlap with one another.

VI. SUPPLEMENTARY REFERENCES

[1] K. H. Kim, K. Amann-Winkel, N. Giovambattista, A. Sah, F. Perakis, H. Pathak, M. L. Parada, C. Yang, D. Mariedahl, T. Eklund, T. J. Lane, S. You, S. Jeong, M. Weston, J. H. Lee, I. Eom, M. Kim, J. Park, S. H. Chun, P. H. Poole, and A. Nilsson, Science **370**, 978-982 (2020).

[2] A. Shalit, F. Perakis, and P. Hamm, J. Phys. Chem. B **117**, 15512-15518 (2013).

[3] A. Shalit, F. Perakis, and P. Hamm, J. Chem. Phys. **140**, 151102 (2014).

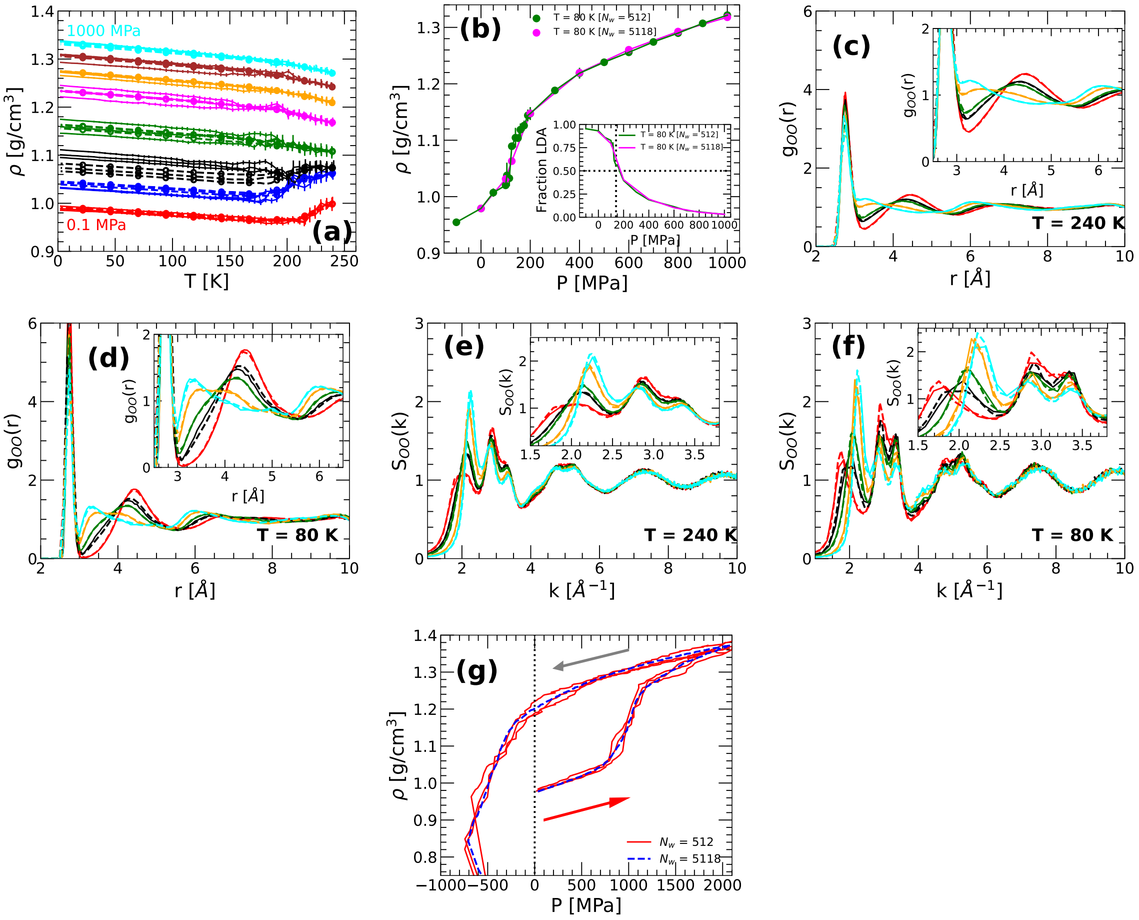

FIG. S1. Comparison of MD simulations results obtained for systems composed of $N = 512$ [solid lines, taken from Figs. 1 and 3(a) of the main manuscript] and $N = 5118$ [dashed lines] water molecules (cooling rate $q_T = 10$ K/ns; compression/decompression rate $q_P = 100$ MPa/ns). (a) Density of q-TIP4P/F water during isobaric cooling at $P =$ 0.1, 100, 125, 200, 400, 600, 800 and 1000 MPa (dots, bottom-to-top) from $T = 240$ down to 0 K; three independent runs are included for each pressure. Size effects are negligible within the fluctuations among the three runs performed at a given pressure and N. Fluctuations at a given cooling pressure, among the corresponding three independent runs, are reduced with increasing N – except for $P = 125$ MPa and in the proximity of the LLCP in q-TIP4P/F water. (b) Density of the IA at $T = 80$ K produced during the isobaric cooling runs included in (a). Inset: fraction of LDA molecules in the IA states included in the main panel. (c)(d) OO RDF of water in the equilibrium liquid (c) and amorphous ice states (d) included in (b). (e)(f) Structure factor $S(k)$ of the liquid and amorphous ices included in (c) and (d), respectively. (g) Isothermal compression-induced LDA-to-HDA transformation at $T = 80$ K followed by the isothermal decompression of HDA from $P = 2000$ MPa to negative pressures ($T = 80$ K). Red lines are from Fig. 3(a) in the main manuscript for the case $N = 512$; dashed lines are from MD simulations with $N = 5118$. In all panels, system size effects are negligible.

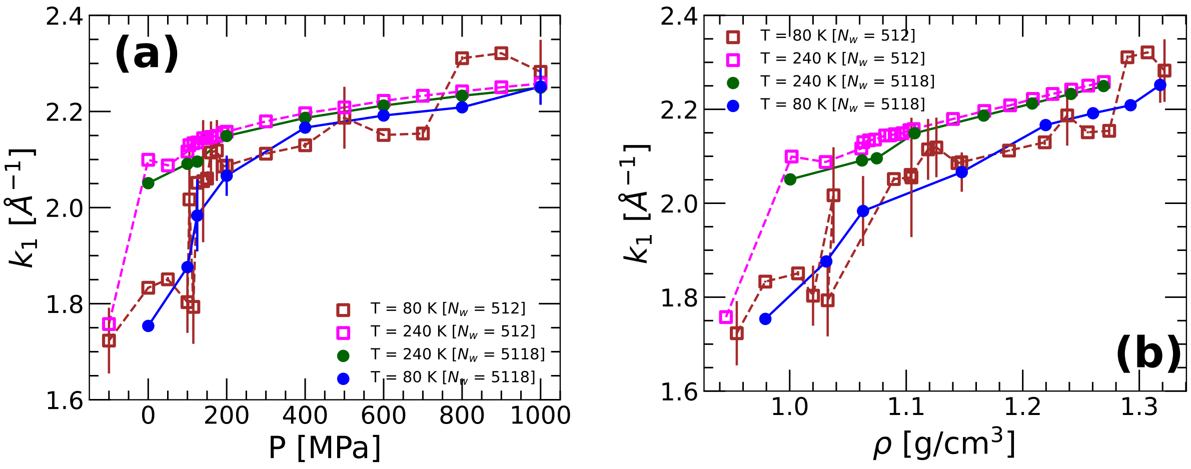

FIG. S2. (a) Location of the structure factor first peak as a function of pressure, $k_1(P)$, of the equilibrium liquid at $T = 240$ K (magenta and green symbols) and the corresponding IA at $T = 80$ K (brown and blue symbols) [from Figs. 1(e)(f) of the main manuscript, and Figs. S1(e)(f)]. (b) $k_1(\rho)$ as a function of the density. The experimental values of k_1 for LDA and HDA are approximately 1.7 and 2.1 Å^{-1}, respectively [1]. At each pressure, we perform three independent simulations. The three independent values of k_1 are then averaged (symbols), and the corresponding standard deviations are represented by the error bars.

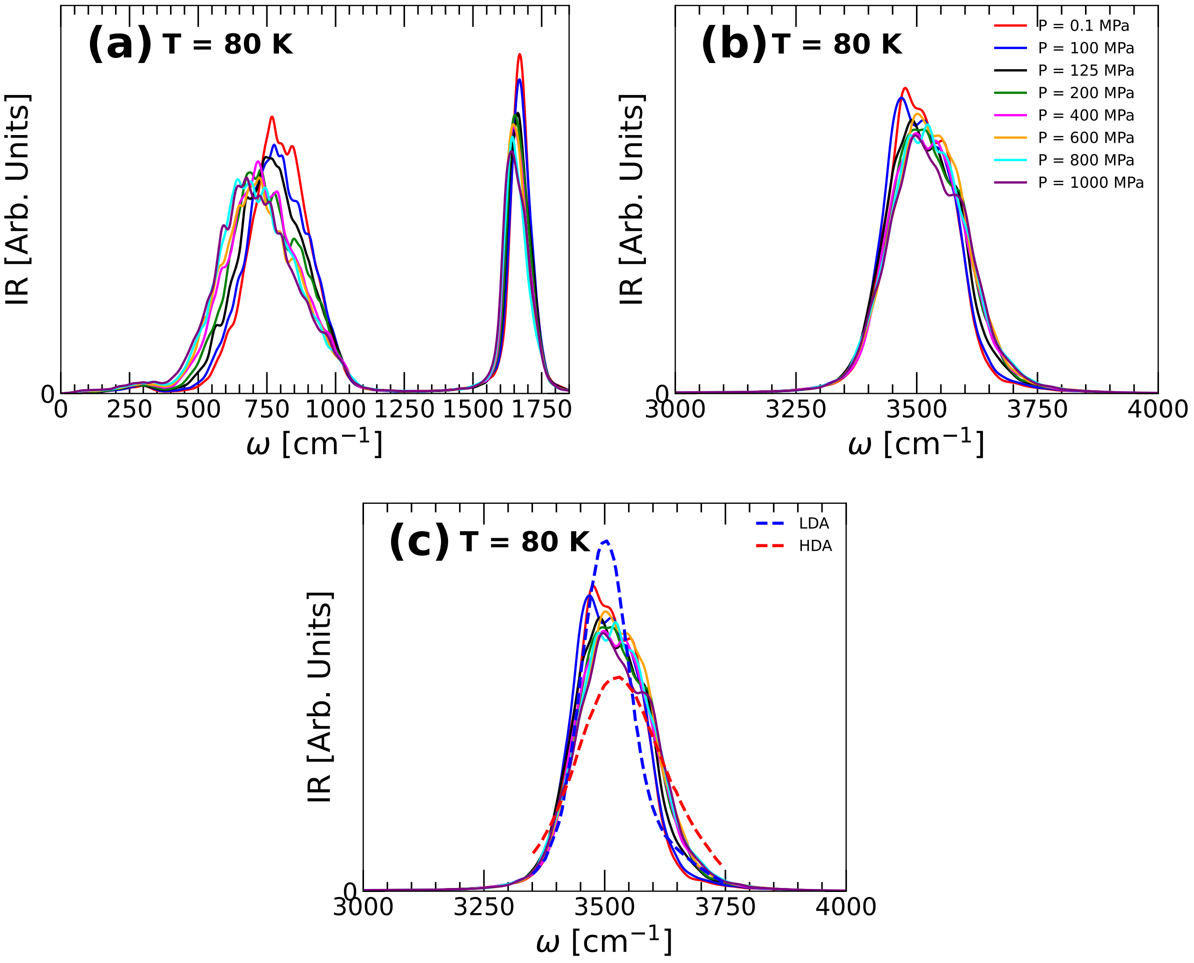

FIG. S3. Infrared spectra (IR) of IA obtained from isobaric cooling simulations at T = 80 K for frequencies (a) $0 < \omega < 1800$ cm^{-1}, covering the librational ($\omega \approx 800$ cm^{-1}) and bending ($\omega \approx 1600$ cm^{-1}) mode frequencies [magnifications of Fig. 1(h) in the main manuscript]. (b) Same as (a) for frequencies $\omega > 3000$ cm^{-1}, corresponding to the OH stretching vibrational modes. Increasing the pressure of the system shifts the librational and bending modes to smaller frequencies and increases the OH stretching mode higher frequencies. (c) OH stretching vibrational modes for the IA compared to the experimental IR spectra for LDA (blue dashed line) and HDA (red dashed line) from Refs. [2, 3]. For a better comparison, the experimental IR spectra are shifted by $\delta\omega = +200$ cm^{-1}. The IR spectra peak shifts towards higher frequencies as the system evolves from LDA to HDA.

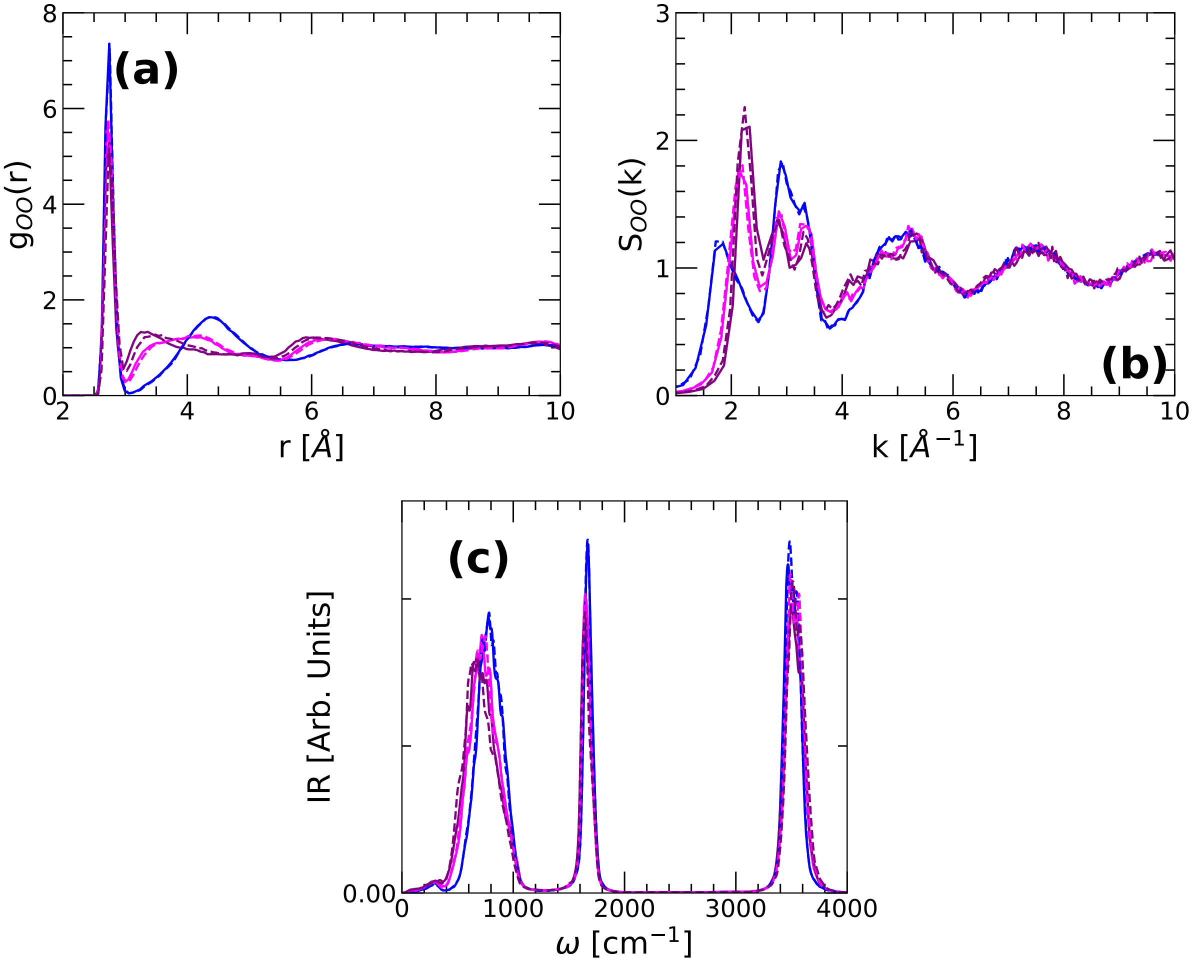

FIG. S4. (a) OO radial distribution function, (b) structure factor $S(k)$, and (c) infrared spectra of the IA at $T = 80$ K. The solid lines are the results for the IA at their preparation pressure $P = 100$ (blue), 400 (magenta), and 1000 MPa (maroon); the dashed-lines are for the IA after their further isothermal decompression ($T = 80$ K) to $P = 0.1$ MPa. The structural changes in the IA induced by the decompression process are minor, if any.

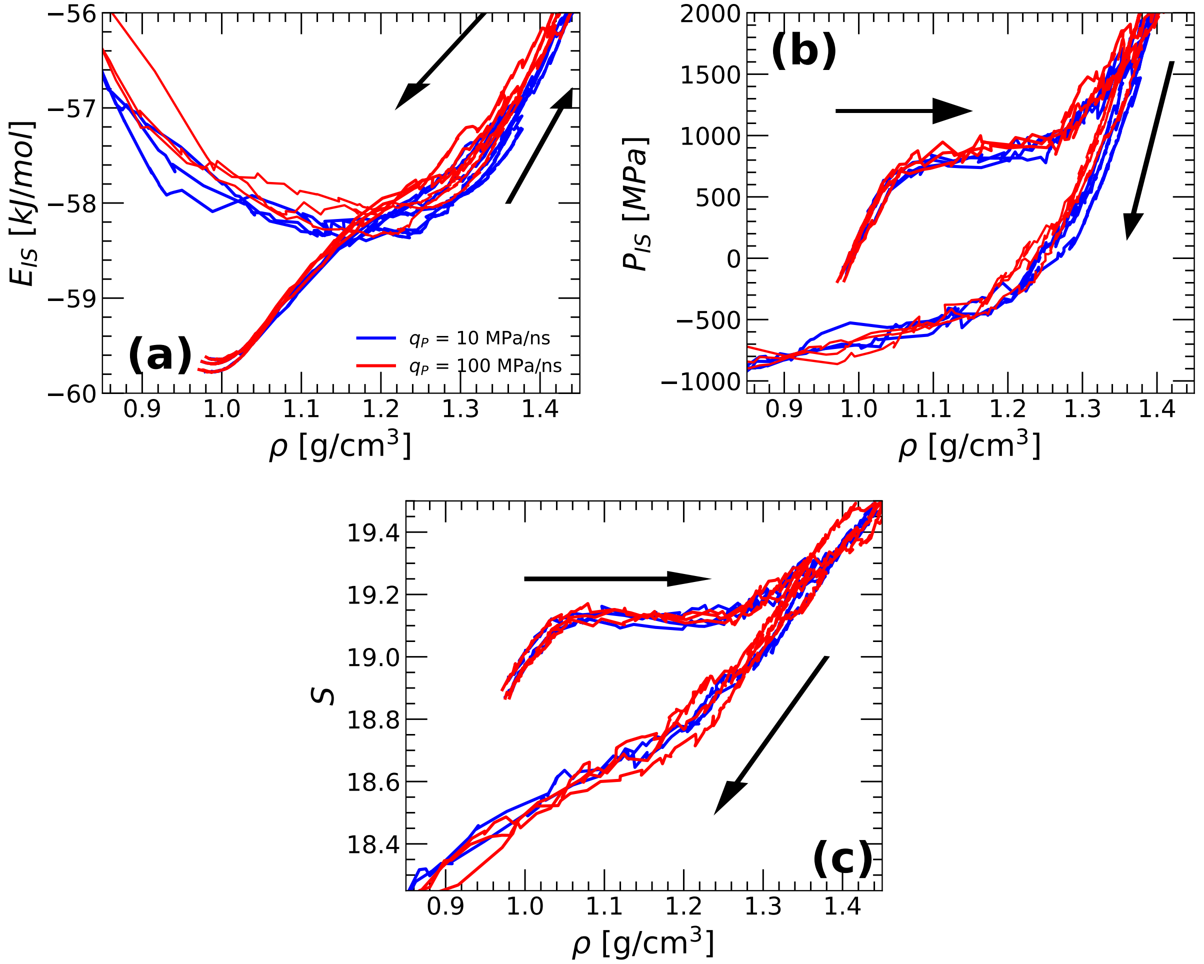

FIG. S5. Average (a) IS energy, (b) IS pressure, and (c) basin shape function of the amorphous ices accessed by the system during the pressure-induced LDA-HDA transformation at $T = 80$ K. Red lines are obtained with a compression/decompression rate $q_P = 100$ MPa/ns (from Fig. 3(a) of the main manuscript); blue lines are for the case of $q_P = 10$ MPa/ns. The results obtained with both compression/decompression rates are practically identical.
